# New Products

**DOI:** 10.1155/S1463924679000651

**Published:** 1979


### New Products

### Liquid analyser

Rank Hilger have introduced a multi-channel liquid analyser, the Chemispek, which uses the principal of continuous flow. It has been designed primarily for the clinical market. Early instruments in production are for electrolyte analysis and a range of other routine tests.

The instrument is modular in construction and is expandable from a two-channel system up to 12 channels, with the addition of twin channel units. Each twin channel will perform either two separate analyses or, where necessary, one analysis with associated blank correction. The units include a peristaltic pump feeding both channels, a cartridge base with heater control modules, two chemistry trays and a two channel photometer. There can be up to two heating baths per channel and one dialyser per channel.

For measurement of sodium potassium, a flame photometer is used with its own associated diluter and DA converter to allow an analogue recorder output. Data presentation is through a purpose-built microprocessor based data handling system; each channel is monitored by a pen recorder output.

*Rank Hilger, Westwood, Margate, Kent CT9 4JL, UK.*

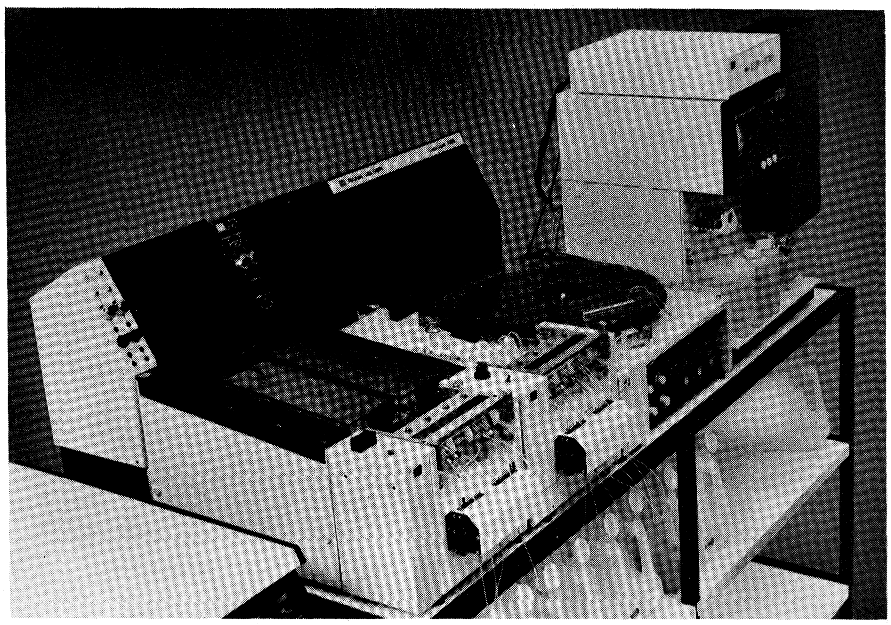

*The Rank Hilger Chemispek liquid analyser*

### Twin channel titrator

The Radiometer microprocessor controlled titrator, a two channel system, has been developed to enable the operator to run one sample on one burette, whilst preparing another sample for another burette, minimising waiting time on the part of the operator and speeding up the analysis time. One microprocessor can control two burettes and titration assemblies, both of which may perform different titrations or the same titration as desired.

*Radiometer A/S, Dk-2400 Kopenhagen NV, Emdrupvej 72, Denmark.*

### Clinical analyser

The Bausch and Lomb 2100 clinical analyser can perform an end-point or kinetic-rate test either singly, or in repeat mode. An adjustable volume peristaltic pump aspirates the sample to a Peltier thermoelectric microflowcell in the specially designed clinical spectrophotometer. The microprocessor will collate the information and use the programmable kinetic rate test after a preset delay to allow time for temperature equilibration.

*Bausch and Lomb, Analytical Systems Div, 820 Linden Ave, Rochester, N.Y., USA 14625.*

### Data logger

A modular microprocessor-based data acquisition and analysis system aimed at the industrial control market has been developed by the Golden River Company Ltd. The system, the Mk4 programmable data logger/controller, provides facilities for data collection, event recording, monitoring and logging as well as real time processing to effect control. Special purpose application programs may be developed in the languages PASCAL and MICRO-FORTH, or in assembler or machine code and held on PROMs. The main application areas are in sequence batch processing, production logging and automatic testing.

The logger is based on the RCA CDP1802 CMOS microprocessor and has power requirements of the order 15 to 50 mA provided by a rechargeable nickel cadmium battery unit. This makes the device suitable for remote logging applications where mains power is not available.

Seventeen interchangeable hardware modules are available which allows the systems to be made to suit individual requirements. Modules include the GR 0405 single board processor, RAM and PROM boards with up to 64k bytes capacity for storage of program and data, up to 8k byte EPROM boards, a manual I/L port, 16 channel analogue output and input boards and a para I/O board providing four latched eight bit output ports, two ports and four asynchronous RS232 compatible I/O ports. Standard interfaces ensure compatibility with a variety of peripheral equipment. Software products available as an aid to programming include the GRUTIL utility program on PROM, a PASCAL compiler a Micro-Forth interpreter/compiler and an assembler/editor.

A comprehensive library of user programs as well as a full software development service and a turn-key service are available.

*Golden River Company Ltd, Telford Road, Bicester, Oxon, OX6 OUL, UK.*

### Computing integrator

Spectra-Physics' Autolab Division have announced the introduction of the SP 4100 Computing Integrator. This instrument features automatic integration, built-in BASIC programming, an X-Y plotting capability, intelligent dialogue, and a range of pre-programmed data reduction methods. It has been designed primarily for use with gas or liquid chromatography systems and is microprocessor controlled. It has an alphanumeric keyboard, a full width LED display, and a printer-plotter output. The BASIC programming capability allows the user to modify existing data reduction methods, to develop special reports or applications, and to reprogramme the standard instrument ROM software, if required.

Many data reduction methods used in chromatography are preprogrammed in permanent memory, including area per cent, area normalisation, external standard, and three variations of internal standard, together with special calculations such as statistics and linear/non linear multi-level calibration. In addition to the keyboard, there are 15 function keys labelled in chromatographic terms. These can all be redesignated by the user to suit his specific requirements.

The unit can be interfaced to the SP 4000 multi-channel data system, which extends the capabilities of both systems.

*Spectra-Physics Ltd, 17 Brick Knoll Park, St. Albans, Herts AL1 5UF, UK.*

### Process control

Turnbull Control Systems Ltd have extended their supervisory control based on Matric 6000 modules. It now includes a single-loop microprocessor-controller, the Matric 6350. Each Matric 6350 controller has a microprocessor which stores the information that gives each unit individual characteristics in the system and is also available to the operator to enable parameter changes to be made as required.

The processor-controller unit is solid-state and is set up by a hand-held terminal with which it is programmed for its particular function in the system. The terminal can be plugged in at any time to permit control parameters to be changed. The controller is compatible with the TCS range of Matric 6000 modules, upon which both systems are based.

*Turnbull Control Systems Ltd, Mulberry Lane, Goring-by-Sea, Worthing, Sussex, UK.*

### Applications program

An applications program, SEARCH, from Perkin-Elmer simplifies the process of interpretation and identification of infrared spectra by addressing one of the difficult operations associated with the IR field. It has been designed for use with their infrared data station and interprets a spectrum in terms of the molecular functional groups present in the unknown molecule. A library of 2000 spectra of selected compounds accompanies the software on one side of a micro disc. The system can be adapted by adding spectra to the library.

Data may be entered manually or by placing the sample in the infrared instrument which is interfaced to the data station. The program will then acquire and process the data automatically.

*Perkin-Elmer Ltd, Post Office Lane, Beaconsfield, Bucks, UK.*

### Acoustic microscope

CAMECA has become the sole European distributor for a scanning laser acoustic microscope, SLAM. The Sonomicroscope, manufactured by Sonoscan Inc. USA, offers a method of non-destructive high resolution investigation of materials. Transmission and interference images provide information about the elastic properties and density of the specimen. Typical applications are the development and quality assurance of ceramic capacitor chips, flaw detection in hot pressed silicon nitride and the detection of any inclusion or bonding fault inside optically opaque materials.

*CAMECA, 103 bd Saint-Denis B.P.No.6, 92403 Courbevoie Cedex, France.*

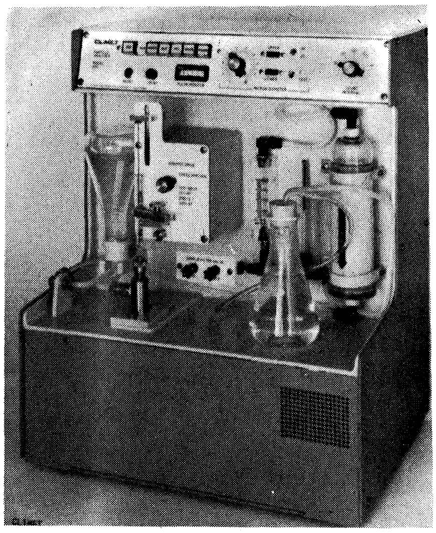

*Climet liquid particle analyser*

### Liquid particle analyser

Climet Instruments have introduced a liquid particle analyser, the Model CI-220 liquid particle analyser. It incorporates an optical sensor which detects and measures individual particles with accuracy. Applications include pharmaceutical manufacturing, cleanliness standards, low viscosity fluids, chemical and biological studies, water pollution studies, food, beverage and dairy product processing and liquid dispensable solids.

*Wessex Electronics Ltd, 114-116 North Street, Downend, Bristol BS16 5SE, UK.*

### Analyser systems

Two new self-calibrating automated stat/routine analyser systems, Astra TM4 and Astra TM8 incorporate microprocessor control and monitoring for fast testing of electrolytes and routine chemistries. There are six chemistry modules currently available, all based on proven analytical methodologies: glucose by oxygen-rate sensing, blood urea nitrogen or urea by rate conductivity, creatine by rate colorimetry, sodium and potassium by ion-selective electrodes, chloride by coulometric titration and carbon dioxide by rate pH. Laboratories can customise either instrument to satisfy specific requirements by the selection of required modules.

The Astra TM4 is capable of running five chemistries and the TM8 nine chemistries. Up to 80 samples can be programmed in advance or during a chemistry run without loss of results using the microprocessor. Computer/operator communication on the TM4 is achieved by a printer and on the TM8 by means of a video screen or printer. Display and printout identify the sample, the patient test requested, the reference ranges in addition to flagging abnormal test results.

Step-by-step computerised diagnostic routines displayed on the video screen or printer simplify the servicing of the systems. The built-in microprocessor constantly monitors instrument status and automatically warns of malfunctions, identifying the location and nature of the trouble for the operator.

*Beckman RIIC Ltd, Turnpike Road, Cressex Industrial Estate, High Wycombe, Bucks, UK.*

### Autosampler

The microcomputer controlled autosampler, Model AS-40, for furnace atomic absorption is now available from Perkin-Elmer. It is designed for use with their HGA-500 and the new HGA-400 heated graphite furnaces. The autosampler is offered in two versions, one configured for the automated Model 5000 instrument, and the other for use with all other Perkin-Elmer microcomputer controlled atomic absorption instruments. It can automatically present up to 35 samples to the instrument for analysis and activate the 'read' cycle of instruments with microcomputer electronics. Sample volume is also microcomputer-controlled and is variable from 5 to 99 $\mu$l. The instrument features automatic method of additions using two procedures and automatic matrix modification. Automatic calibration of the Models 5000, 703, 603, 560 or 450 is built-in. Recalibration or reslope may be performed as many as three times during operation.

*Perkin-Elmer Ltd., Post Office Lane, Beaconsfield, Bucks HP9 1QA, UK.*

### Multiwavelength photometer

A UV/visible photometer, the 860 absorbance detector from Du Pont, is designed for high sensitivity detection of chromatographically separated components that absorb light at the mercury emission lines in the 254 to 546 nm wavelength region of the electromagnetic spectrum. Under the proper measuring conditions, it is claimed that sample components can be detected at picogram levels with this device. The photometer uses optical filters and a low pressure mercury lamp source with a phosphor-coated tip. A two-position mounting permits easy conversion from 254 to 284 nm.

The flow-cell design permits sensitivities to 0.002 absorbance units full scale (AUFS). Wide dynamic range with linear detector response from 0.002 to 2.0 AUFS permits optimum sensitivity and quantitative analysis. Sample absorbance is continuously displayed on a digital meter for visual monitoring and recording.

*Du Pont (UK) Ltd, 64a Wilbury Way, Hitchin, Herts SG4 OUR, UK.*

### Computing integrator

Pye Unicam have reached an agreement with Spectra Physics to market their SP4100 computing integrator in the UK. The SP4100 is fully compatible with Pye Unicam's range of Gas and Liquid Chromatographs. It is microprocessor controlled, has a full Alphanumeric keyboard and LED display and an integral printer plotter. BASIC is standard in the SP4100 with extensive built-in programming which can be implemented with just a few key strokes.

Built in BASIC programming capability allows the user flexibility to modify an existing data reduction method, format his own report or develop other custom applications. The user can modify, through a programme overlay, the BASIC preprogrammed methods in ROM.

The plotter prints at 24 characters per second in black on white and can 'chart reverse', thus enabling it to do X-Y plotting such as graphing a multi level calibration. It has a full Alpha Numeric keyboard with both upper and lower case characters plus 15 function keys labelled in familiar chromatography terms. There is an intelligent interactive dialogue and automatic dynamically optimised integration parameters.

*Pye Unicam Ltd, York Street, Cambridge CB1 2PX, UK.*

### Sample concentrator

The evaporation of solvents from samples can be a time consuming operation in the preparation of samples for analysis. The Techne SC-3 sample concentrator can be used for any multi-evaporation requirement of solvents in test tubes or centrifuge tubes. It is particularly useful when using thermolabile substances where it is possible to effect adequate evaporation rates at lower temperatures than with heating alone. Any size of standard test tube in the range of 6 mm to 25 mm can be used with the unit which can handle up to 90 of the 6 mm size tubes. Gas, $CO_2$ or $N_2$ is introduced into the test tube by a multi-outlet gas reservoir via hypodermic needles.

*Techne Cambridge Ltd, Duxford, Cambridge, CB2 4PZ, UK.*

### UV Detector

Schoeffel Instrument Division of Kratos have recently introduced an ultraviolet detector for HPLC. The detector, the model SF 740, employs a deuterium lamp and can therefore perform absorption measurements at any wavelength between 200 and 380 nm without changing the lamps. Quick-change filter cassettes isolate the wavelength of interest. Standard filters available include 200, 215, 240, 254 and 280 with other wavelengths available on special order. The use of shorter UV wavelengths has been found useful in the detection of amino acids, peptides, many carbohydrates, and many other compounds.

The detector features an easily removed and cleaned flowcell with a small 10 $\mu l$ volume. Nine calibrated absorbance ranges from 0.005 to 2.0 AUFS are push-button selectable. Noise in this single beam reference compensated detector is less than $1 \times 10^{-4}$ AU. The SF 740 can be interfaced with any liquid chromatography.

*Kratos Inc, Schoeffel Instrument Division, 24 Booker Street, Westwood, NJ 07675, USA.*

### Blood chemistry analyser

An automatic blood chemistry analyser, the Hitachi Abca, was displayed for the first time in the UK at the International Clinical Chemistry Congress in Brighton by LKB. The analyser is capable of selective profiles of up to twelve chemistries but follows the philosophy of discrete analysis. It embraces a reaction cup laundry system and is simple to operate because of its microprocessor control and reporting system.

*LKB Clinicon Systems Ltd, Bell Lane, Lewes, East Sussex BN7 1LG, UK.*

### Notes for Contributors

### Presentation of manuscripts

Manuscripts should be typed (double-spaced) on one side of the paper only and with generous margins. The title should be brief and informative avoiding the word "new" and its synonyms. The full list of authors with their affiliations and full address(es) should appear on the title page. On a separate sheet an abstract of no more than 150 words is required. This should succinctly describe the scope of the contribution and highlight significant findings or innovations. It should be written in a style which can easily be translated into French and German.

*The Concise Oxford Dictionary* and Fowler's *Modern English Usage* (both published by Oxford University Press) should be used as the standard for spelling and grammar. Abbreviations should be limited to those generally recognised, or where a frequently occuring term is abbreviated it should, in the first instance, be explained thus "flow injection analysis (FIA) ..." and the abbreviation used thereafter. Abbreviations, for standard measures and units should follow SI recommendations. There are various publications giving guidance on the use of SI units.

References should be indicated in the text by numerals following the author's name, i.e. Skeggs [6]. On a separate sheet of paper, list all references in numerical order thus:

[6] Skeggs, L.T., *American Journal of Clinical Pathology,* 1959, **28**, 311.

Note that journal titles are given in full. Where there is more than one author, the form Foreman et al. should be used in the text but all authors should be named in the list of references. When reference is made to a chapter in a book the reference should take the following form:

[7] Malmstadt, H.V. in "Topics in Automatic Chemistry" Ed. Stockwell P.B. and Foreman J.K. 1978 Horwood, Chichester, pp. 68-70.

Only work which has been published or has been accepted for publication should be cited. Avoid the citation of documents which are subject to restricted circulation, patent literature, unpublished work and personal communications. The latter can be mentioned in the text in parenthesis.

To illustrate a paper line diagrams are preferred to photographs. Photographs should only be used when they significantly add to the discussion. Diagrams, charts and graphs should be carefully drawn in black ink on stout card or heavy quality tracing paper. Most illustrations are reduced for publication; to allow for this originals should be between 16 and 36 cm wide (the depth must not exceed 50 cm). The lettering of diagrams should be sufficiently clear to withstand reduction. Except in the case of proper names, all lettering should be in lower case print. If photographs are used they must be supplied in the form of clear, unmounted, glossy, black and white prints. "Instant" photographs are not normally acceptable. All illustrations must be identified on the reverse showing the figure number and the author's name.

Each illustration should have a fully explanitory caption. Captions should be typed together on a separate sheet of paper; they must *not* be an inseparable part of the illustration.

